# The application of scanning near field optical imaging to the study of human sperm morphology

**DOI:** 10.1186/s12951-014-0061-5

**Published:** 2015-01-16

**Authors:** Laura Andolfi, Elisa Trevisan, Barbara Troian, Stefano Prato, Rita Boscolo, Elena Giolo, Stefania Luppi, Monica Martinelli, Giuseppe Ricci, Marina Zweyer

**Affiliations:** IOM-CNR, Area Science Park, Basovizza, Trieste, Italy; Department of Life Sciences University of Trieste, Trieste, Italy; A.P.E. Research Srl, AREA Science Park, Basovizza, Trieste, Italy; Institute for Maternal and Child Health, IRCCS Burlo Garofolo, Trieste, Italy; Department of Medicine, Surgery and Health Sciences, University of Trieste, Trieste, Italy

**Keywords:** Spermatozoa, Scanning near-field optical microscopy, Morphology

## Abstract

**Background:**

The morphology of spermatozoa is a fundamental aspect to consider in fertilization, sperm pathology, assisted reproduction and contraception. Head, neck, midpiece, principal and terminal part of flagellum are the main sperm components to investigate for identifying morphological features and related anomalies. Recently, scanning near-field optical microscopy (SNOM), which belongs to the wide family of nanoscopic techniques, has opened up new routes for the investigation of biological systems. SNOM is the only technique able to provide simultaneously highly resolved topography and optical images with a resolution beyond the diffraction limit, typical of conventional optical microscopy. This offers the advantage to obtain complementary information about cell surface and cytoplasmatic structures.

**Results:**

In this work human spermatozoa both healthy and with morphological anomalies are analyzed by SNOM, to demonstrate the potentiality of such approach in the visualization of sperm morphological details. The combination of SNOM topography with optical (reflection and transmission) images enables to examine typical topographic features of spermatozoa together with underlying cytoplasmic structures. Indeed the head shape and inner components as acrosome and nucleus, and the organization of mitochondria in the midpiece region are observed. Analogously for principal tract of the tail, the ridges and the columns are detected in the SNOM topography, while their internal arrangement can be observed in the corresponding SNOM optical transmission images, without requiring specific staining procedures or invasive protocols.

**Conclusions:**

Such findings demonstrate that SNOM represents a versatile and powerful tool to describe topographical and inner structural details of spermatozoa simultaneously. This analysis could be helpful for better characterizing several morphological anomalies, often related to sperm infertility, which cannot be examined by conventional techniques all together.

**Electronic supplementary material:**

The online version of this article (doi:10.1186/s12951-014-0061-5) contains supplementary material, which is available to authorized users.

## Background

Conventional semen parameters such as sperm concentration, motility and morphology are generally used to assess male fertility [1-3]. However, a significant percentage of males with normal semen parameters, according to WHO guidelines [4], fails to conceive [5]. In these cases, the presence of ultrastructural defects could be hypothesized [6-11]. Several morphological studies have been carried out to define the normal form of sperm (see reference [9] for review) and the last WHO semen manual provides objective criteria to assess the sperm morphology [4]. However, there are many factors that may influence the results of the morphology assessment, including the technician’s concept of the definition of normality [12,13] and the staining procedures [14,15]. The most common technique to assess sperm morphology is conventional optical microscopy (OM), usually performed on fixed and stained specimens. In this case, sample preparation is rather easy but the resolution is limited to micron resolution (0.2 μm: Abbe diffraction limit). Moreover, it has been shown that different staining techniques may cause not uniform changes in sperm head dimensions [15]. To reduce the subjectivity of sperm morphology assessment, computer-assisted sperm morphometry analysis (CASMA or ASMA) have been developed in the ‘90s [16,17]. Subsequently, this analysis has been improved by using special objectives for relief contrast observation [18]. A further progress has been achieved by combining fluorescence microscopy and image analysis with open-access software [19,20]. This method allows automatic determination of sperm morphometry in a reduced time [19,20]. To avoid artefacts induced by fixation and staining, a new optical technique (Trumorph system) has been recently described [21]. This technique allows the analysis of living unmodified spermatozoa that are immobilized in narrow chambers and examined by negative contrast phase microscopy [21]. In the last decades, other fundamental studies have been carried out by transmission and scanning electron microscopies (TEM and SEM), which have enabled to detect ultrastructural details and have provided a huge amount of information concerning morphological organization of spermatozoa [22-25]. However, SEM imaging usually needs harsh sample preparation as drying at critical point, metal coating and vacuum environment (during coating procedure and measurements) that may modify some structural features of cells. Analogously, complex preparation of specimens (as resin embedding and sectioning by ultramicrotomy) are required for performing TEM imaging, which provides two-dimensional view of cell internal arrangement. Recently scanning probe microscopy (SPM) techniques have been exploited to improve the knowledge of spermatozoa morphology. Particularly, atomic force microscopy (AFM) has allowed investigating the topography of spermatozoa with nanometric resolution and without needing invasive sample preparation [26-29]. Moreover, either fixed or living cells can be imaged by this technique. Among scanning probe microscopies (SPM), scanning near-field optical microscope (SNOM) has been demonstrated to be a powerful and versatile tool for the observation of biological samples [30,31]. As in AFM, a sharp probe scans the sample surface to generate a topographic image with similar resolution and sensitivity [32]. The SNOM probe consists of a tapered optical fibre that scans the surface and acts as a point light source with a subwavelength-aperture at the terminal part [32,33], which creates a near-field light that decays exponentially with the distance of 100 nm and carries highly localized information [34]. As result, subdiffraction resolution optical images (reflection, transmission, back-reflection and fluorescence) and high-resolution (nanometer level) topographic image are simultaneously generated. Such technique has been successfully applied for the investigation of cell membranes at molecular level [35-38], subcellular structures [39,40], thin-sections [41] and chromosomes [42]. In the present work, we exploit the advantage of SNOM technique to describe simultaneously different superficial and internal morphological details of human spermatozoa from normozoospermia and teratozoospermia, without needing invasive or expensive sample preparation.

## Results and discussion

Morphological analysis of the sperm structures provides relevant information, necessary part to clinical data to evaluate the ability to fertilization. Numerous cellular structures characterizing the spermatozoa have been elucidated thanks to SEM and TEM studies at ultrastructural level, and more recently by AFM at nanometer scale. In this study, the potentialities of SNOM have been exploited to investigate the morphology of fixed human spermatozoa deriving from samples of patients with normozoospermia and teratozoospermia.

**Figure 1:**
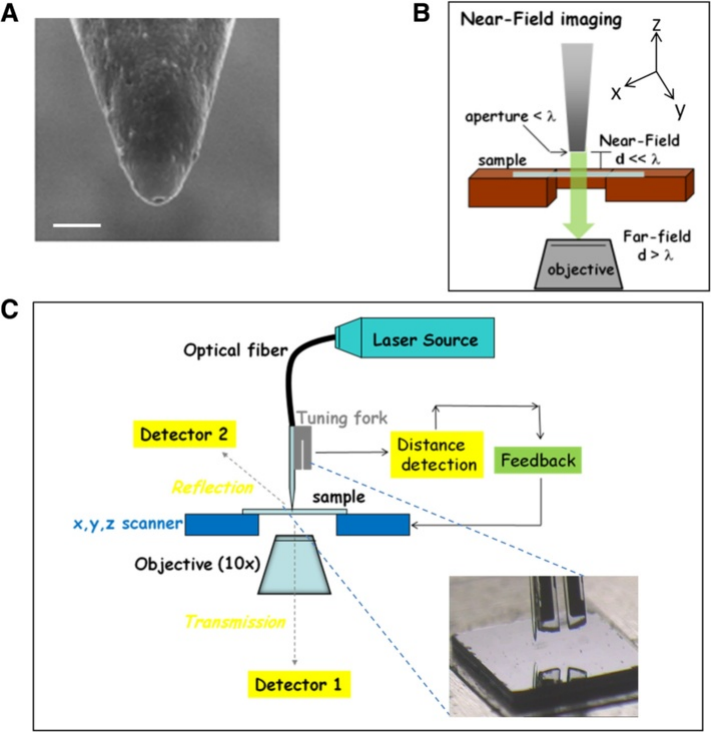
**A schematic representation of SNOM instrumentation and working principle. (A)** SEM image of a SNOM probe: apical part of a metal coated tapered optical fibre with aperture at the end (bar 200 nm); **(B)** the small aperture of the optical fibre is held in close proximity of the sample surface and the local light interaction creates near-field used to reach high resolution, overcoming the optical diffraction limit; **(C)** the position of fibre, the location of reflection and transmission detectors as respect to the scanned sample are shown.

SNOM microscopy offers many distinct advantages over other approaches: it is non-destructive and the sample preparation is simple and inexpensive. SNOM technique exploits the properties of the evanescent field that exists only near the surface, between the probe (optical fibre) and the sample (Figure 1). The probe consists of a single mode tapered optical fibre metal coated with an aperture having a diameter of 50–100 nm at the end of the fibre (Figure 1A). This small aperture, brought at nanometer distance (about 10 nm) from the sample, creates an evanescent component of the incident light whose intensity decays exponentially and allows obtaining optical images with a resolution that overcome the diffraction limit (λ/2) (Figure 1B). The fibre, positioned within few nanometers from the sample surface, is scanned parallel to the surface and the x,y,z data at each point are acquired. The fibre is attached to a tuning fork that oscillates at its resonance frequency; such amplitude can change upon interaction of the probe with the sample during scanning. Hence, a feedback system regulates the probe-sample distance to maintain constant the oscillation amplitude and the probe-sample distance. As a result, an accurate three-dimensional map of the specimen down to the nanometer level can be obtained. This real topographical map of the sample surface, as for AFM, allows measuring the height profile (i.e. vertical size) of the structures investigated [30]. Simultaneously, the light delivered by the fibre that hints the sample locally is scattered (reflected) or adsorbed according to the different optical properties of the structures encountered. The light reflected from sample surface is collected by an upper detector, while that transmitted one (i.e. the light that pass through and not adsorbed by the sample) is collected by a second detector placed below the sample (Figure 1C). These detectors generate SNOM reflection and transmission images simultaneously with high optical resolution in xy axes (<100 nm) higher than conventional optical microscopies (i.e. confocal, phase contrast, differential interference contrast) [39,40]. As result, SNOM allows us to detect simultaneously different signals:

1. Topography: it is an accurate real three-dimensional image of the sample surface. The acquisition of signals in x,y,z axes allows measuring surface details at nanometric level. Differently from traditional microscopies (2D methods), SNOM produces a real 3D topography image, since z dimensions are directly measured point by point on the vertical axis, analogously to AFM images.
2. Optical reflection: the SNOM reflection image derives from local interactions between near field light and the surface of the specimen, in particular is due to the light scattered from a thin layer within 30–100 nanometers below the sample surface (depending on tip aperture). Hence this image provides mainly morphological information regarding structures immediately below the sample surface.
3. Optical transmission: the light transmitted through the whole thickness of the specimen and not adsorbed by the sample produces the SNOM transmission image. In conventional optical microscopy the entire sample is illuminated. On the contrary, in near-field the light interacts locally, producing signals point by point to reconstruct a whole image of the scanned area. Hence, SNOM imaging is quite similar to traditional optical transmission imaging, but its lateral resolution is more than 10 times higher.

SNOM optical reflection and transmission images can put into evidence the organization of cellular structures positioned at cell surface (i.e. membrane and structures immediately below) or internal ones (i.e. cytoskeleton, nucleus, mitochondria). Therefore together with the topography they can provide a complete picture of the cell morphology.

Before SNOM imaging the semen was purified from other cells and agglomerated proteins to reduce the amount of impurities that can deposit on glass cover slip together with sperm cells. This is an important aspect to get high quality SNOM images and avoid that tip gets dirty during scanning the surface. Moreover, the sequence of purification, fixation and drying procedures, used for SNOM imaging, did not cause remarkable cell damaging that could lead to the formation of artefacts.

As a matter of fact, sperm were firstly fixed, then partially dehydrated with alcohol and dried just before imaging. This procedure should minimize the morphological artefacts, since they mainly appear when cells are dried before fixation [43,44].

**Figure 2:**
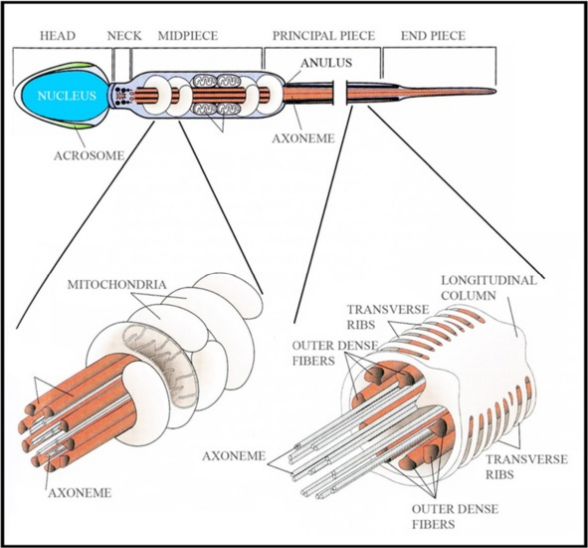
**Spermatozoa morphology.** Sketch illustrating the sperm cellular structures as modified from text book “Embriologia Umana” Morfogenesi - Processi Molecolari - Aspetti Clinici di M. De Felici et al., Piccin. Gentle permission.

To have a clear and immediate picture of the peculiar structures of the spermatozoon, we report in Figure 2 a sketch where the position of such structures is illustrated: head, acrosome, neck, midpiece, anulus, mitochondria that elicoidally surround the axoneme, principal and terminal part of the tail with ultrastructural details (dense fibres and axoneme).

**Figure 3:**
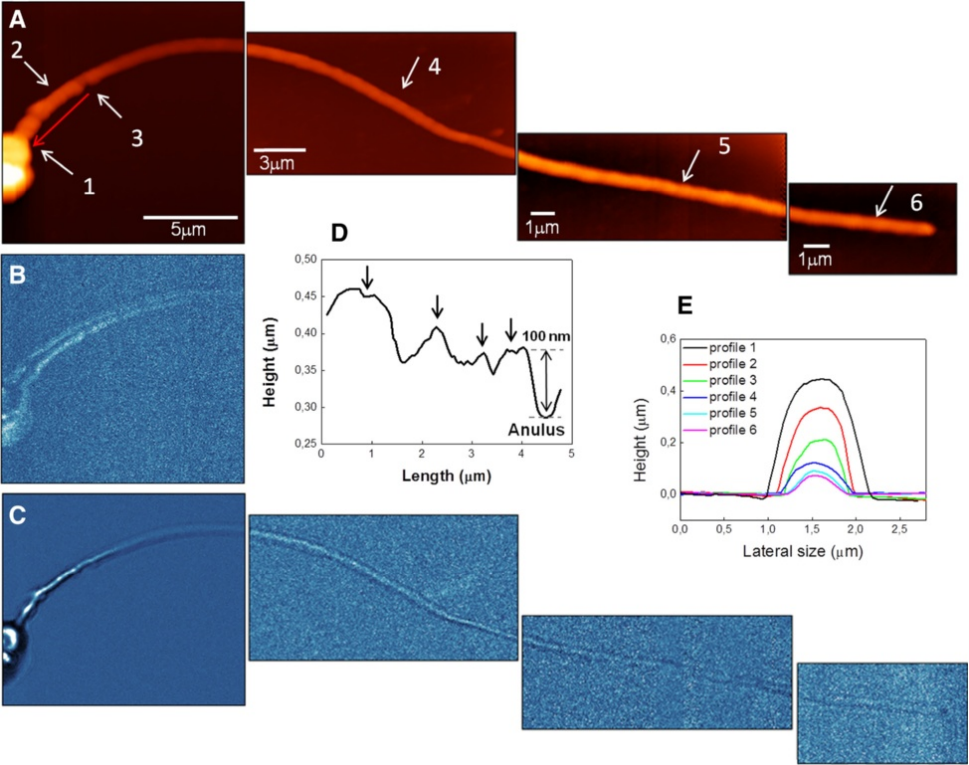
**Reconstruction of a human spermatozoon as imaged by SNOM. (A)** SNOM topography, **(B)** SNOM optical reflection and **(C)** transmission images. **(D)** Longitudinal profile of midpiece along the red arrow, black arrows indicate bumps on cell surface, **(E)** horizontal cross section profiles starting from the initial part of the midpiece up to the end of the flagellum as obtained in the regions indicated by the white arrows 1, 2, 3, 4, 5, 6.

In Figure 3 a reconstruction of a single sperm imaged by SNOM is shown. Figure 3A displays the SNOM topography of the main regions of a sperm cell head, neck, midpiece, anulus, principal and terminal part of flagellum, while SNOM optical reflection and transmission are shown in Figure 3B and C, respectively. The optical images point into evidence that each single region of the sperm has different optical contrast along the cell, likely due to different optical density of the cell structures. SNOM optical reflection images are shown only for the head and principal part, since the rest of the flagellum did not highlight any optical reflection signal.

The analysis of the topographical image is shown in Figure 3D and E. Figure 3D shows a longitudinal profile of the midpiece, between the head and anulus along the red arrow direction of Figure 3A. The profile points into evidence four bumps (indicated by the black arrows), which likely are due to the presence of mitochondria helicoidally surrounding axonema (Figure 2), as also observed in previous AFM work [26]. The total length of this region is found to be 4.5 μm in accordance with the range of values obtained in literature [26]. In Figure 3E horizontal cross section profiles taken on the different part of the sperm are shown (white arrows in Figure 3A). These data display the difference in height of the tail: profile 1, made in the initial part of the midpiece provides height of 450 nm; profile 2, in midpiece region height of 340 nm; profile 3, in the anulus region height of 220 nm. The rest of the flagellum in three points is measured as shown in Figure 3A: profile 4, height of 120 nm; profile 5, height of 90 nm; profile 6, height of 70 nm. These results, in agreement with literature reported values, point out that the scanning of the spermatozoa by SNOM probe does not introduce artifacts and does not damage cell integrity [26,29].

**Figure 4:**
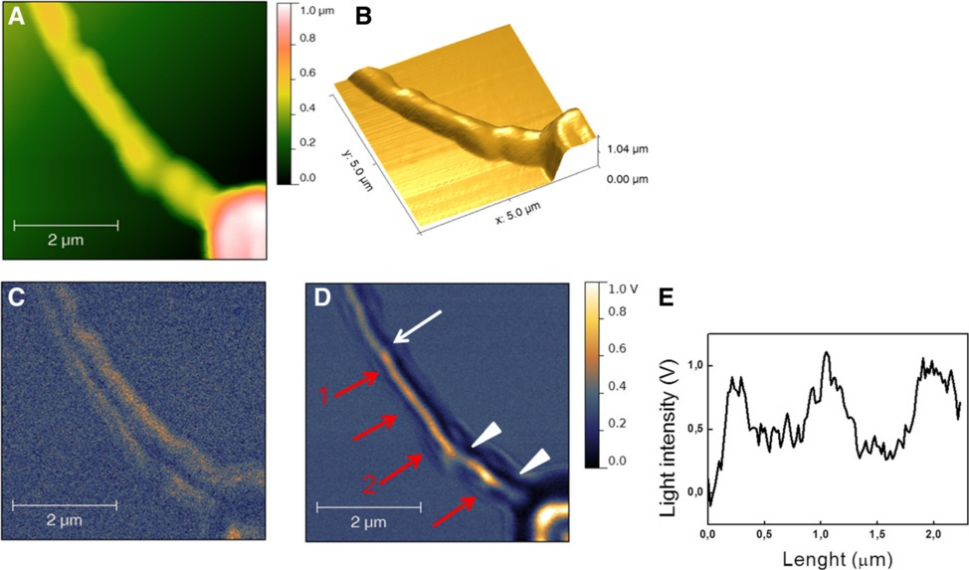
**A zoomed scan area of the midpiece of a human spermatozoon. (A)** SNOM topography, **(B)** 3D representation of the topography, **(C)** SNOM optical reflection and **(D)** SNOM transmission. The white arrow in **(D)** indicates the point where a marked variation in optical contrast along the spermatozoon can be detected. The red arrows in **(D)** indicate the area with ovoid elements where mitochondria are located as illustrated in the sketch of Figure 2. **(E)** longitudinal profile from arrow 1 to arrow 2. Arrowheads indicate additional discontinuities.

Afterward, we closely analyze all parts of the spermatozoon. A representative SNOM image acquired on the midpiece region is shown in Figure 4. In this case the topography (Figure 4A and B) shows structural superficial features at higher resolution respect those previously described. The SNOM optical reflection image in Figure 4C shows differences in optical contrast between the external region of the cell (bright area in the reflection image) and the inner region of the cell (dark in the reflection image). The external bright area mainly involves cell membrane and the cytoplasm region placed immediately beneath. Instead the SNOM optical transmission image (Figure 4D) shows that along the longitudinal axis the midpiece region appears brighter than the rest of the principal part of the spermatozoon. This variation in optical contrast is indicated by the white arrow. In this area some bright ovoid elements can be distinguished (see red arrows) having a length of 0.3-0.5 nm as evaluated by longitudinal profile (Figure 4E), which likely correspond to the mitochondria helicoidally distributed around the axoneme. Moreover, additional discontinuities can be observed as indicated by the arrowheads: the first is in the middle of the midpiece where mitochondria could be not tightly packed, while the second point, close the neck, can identify the different inner organization between midpiece and neck. These data suggest that SNOM optical imaging can allow distinguishing different cytoplasmic structures and components thanks to their different optical density.

**Figure 5:**
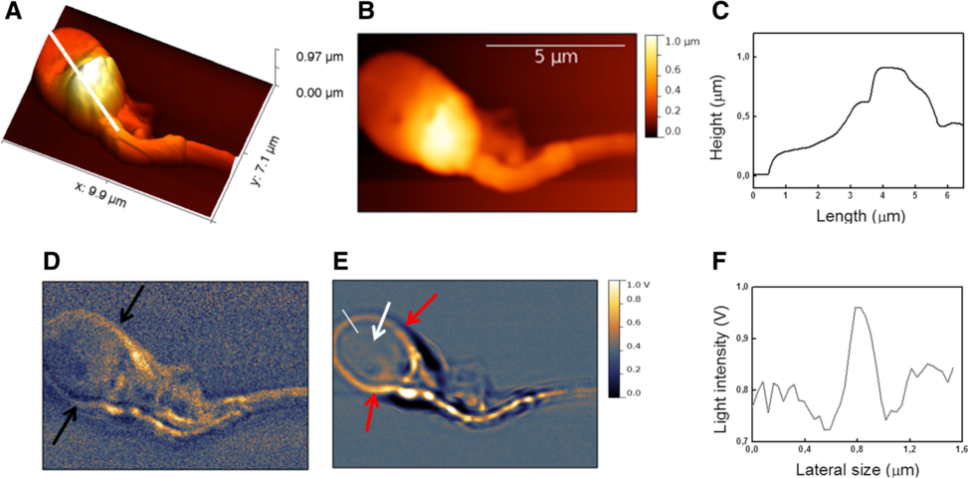
**SNOM image of the head and midpiece of a human spermatozoon.** **(A and B)** SNOM 3D representation and topography, **(C)** longitudinal profile along the head as shown by the white line in **(A)**; **(D)** SNOM optical reflection and **(E)** transmission. In **(D)** black arrows indicate the presence of a faint bright layer around the head, this layer is well-defined and its ends are indicated by the red arrows, while white arrow indicates the nuclear region. In **(F)** horizontal cross section profile along the white line that allows evaluating the width of the bright layer around the head.

In Figure 5, a closer SNOM imaging of the sperm head is shown. Figure 5A and B display the SNOM 3D representation (A) and topography (B) of the head. In Figure 5C the head longitudinal profile provides a length of 5.2 μm, in agreement with the range of values obtained in literature with the size of integral sperm head [15,26,45-47]. Head appears flattened on the frontal part (250 nm on average in thickness) respect to the back (950 nm). The analysis of head shape has been taken into account in previous AFM characterization, performed in physiological condition, where the head shape of healthy sperm is compared to that observed in globozoospermia disease [29], which is a pathology consisting in round acrosomeless head leading to infertility [48,49]. In this case the differences of sperm head profiles in the presence and in the absence of acrosoma is investigated. In our case the front head depression can be ascribed to the partial dehydration process following the fixation procedure. This feature, also observed in other AFM investigation performed in air on fixed spermatozoa, does not cause structural variations of the head [29]. In the SNOM reflection image (Figure 5D) a bright layer surrounding the head can be observed. This bright layer, that appears more evident in the SNOM transmission image (Figure 5E), could be ascribed to region between the plasma membrane and the external nuclear envelope membrane including the acrosome vesicle, which is a cap-like structure partially surrounding the nucleus as indicated in the sketch of Figure 2. In Figure 5F, the two arrows indicate that this bright external layer cover only 2/3 of the head, while the rest is a very dark external part (see also Figure in Additional file 1). The thickness (i.e. width) of this bright layer, evaluated by horizontal cross section profile along the white line, is found to be 230 nm (as evaluated at half width of the maximum height) (see also Figure in Additional file 1). Moreover in these images, immediately below the acrosomal vescicle, the nucleus can be also recognized.

**Figure 6:**
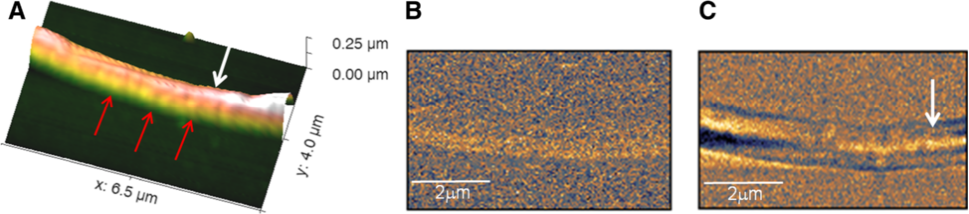
**SNOM image of the principal part of the human sperm tail. (A)** SNOM 3D topography, red arrows indicate the cross circumferential structures one after another along the tail; **(B)** SNOM optical reflection and **(C)** transmission, where the white arrow indicates a bright longitudinal structure.

A high magnification imaging of the principal piece is shown in Figure 6. In Figure 6A red arrows indicate a series of lateral thin bumps that likely can be ascribed to the transverse ribs (sketch of Figure 2). Moreover, in this topography image a longitudinal ridge can be observed (white arrow), which can be attributed to the longitudinal column illustrated in the sketch of Figure 2. This feature can be recognized also in the SNOM transmission image of Figure 6C, where the difference in optical contrast allows following the column position within the tail. In this case the SNOM reflection image (Figure 6B) does not highlight particular differences in optical contrast. These structures may be positioned in the tail likely more deeply than 50–100 nm below the cell membrane, and consequently undetectable by reflection, as already described for SNOM reflection imaging [39].

**Figure 7:**
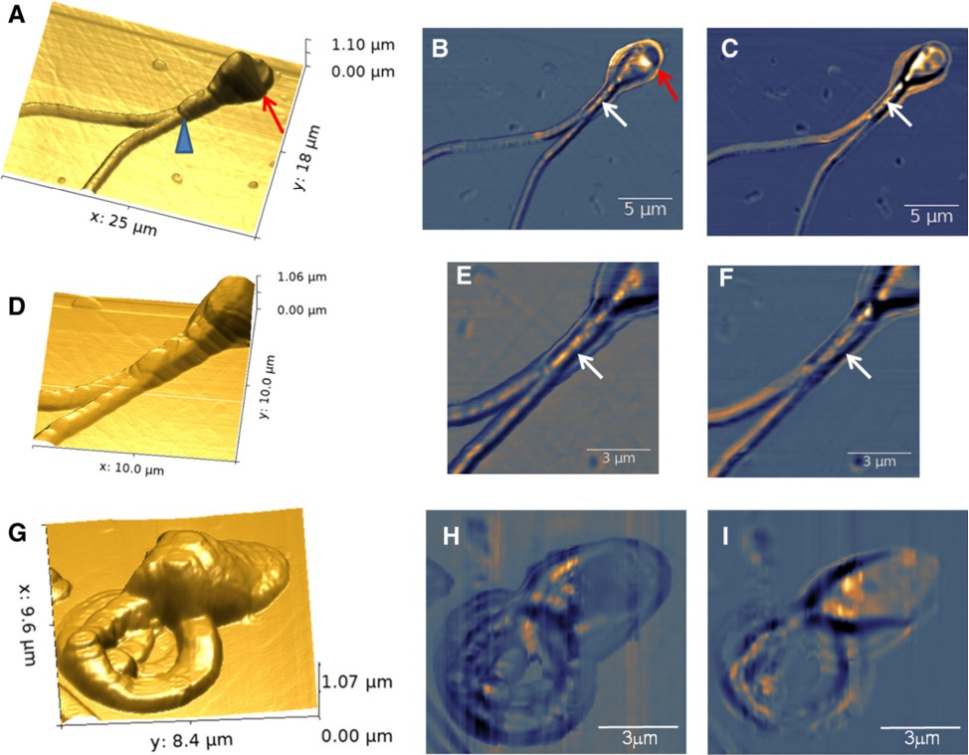
**Human spermatozoa having morphological anomalies as imaged by SNOM. (A)** 3D SNOM topography of two tail spermatozoa: blue arrowhead indicates the end of midpiece, while red arrow indicates the end of post-acrosome area. **(B)** SNOM optical reflection and **(C)** transmission images. **(D)** 3D SNOM topography of a zoomed scan area of midpiece region together with SNOM reflection **(E)** and transmission **(F)** images. **(G)** 3D SNOM topography of a coiled sperm and corresponding SNOM optical reflection **(H)** and **(I)** transmission images.

To understand the potentiality of SNOM technique, imaging is also performed on spermatozoa having morphological anomalies. Figure 7 displays SNOM images acquired on anomalous sperm cells. In this case we take into consideration two sperm anomalies: double tail and coiled tail. In double tail sperm the SNOM topography image shows a head with a reduced acrosome area and a very large post-acrosome area (Figure 7A red arrow). This aspect seems to be also detectable in SNOM reflection image where a limit between a vescicle-like-structure of the head rostral part and the remnant predominantly component can be observed (red arrow of Figure 7B). In the SNOM topography the midpiece is also well distinguishable (Figure 7A and D) and the blue arrowhead highlights the transition to the principal part of the two emerging flagella (Figure 7A). In the corresponding SNOM optical reflection and transmission images the ovoid elements distinguishable in the case of healthy sperm are poorly delineated and the internal part appears to have an irregular organization as compared to healthy one (white arrows Figure 7B, C, E and F). These images suggest that a defective mitochondria organization could be present.

In the case of coiled tail sperm, the SNOM topography (Figure 7G) shows that the midpiece region consists of repetitive structures. Such arrangement can be also detected in the SNOM optical reflection image likely related to an arrangement of the membrane and the layer immediately below (Figure 7H). Whereas in the SNOM optical transmission image, due to the overlapping of the coiled tail, it is difficult to detect and describe single components (Figure 7I).

## Conclusions

In summary, we demonstrate that SNOM technique enables to describe the cell surface and the arrangement of the spermatozoa internal structures both in healthy and anomalous sperm by acquiring topographic and optical (transmission and reflection) images simultaneously. These structural details of spermatozoa can be detected by using rapid, inexpensive and non-harsh procedures for sample preparation. Specifically, the acrosome region located around the head is revealed and evaluated by SNOM optical transmission images. Analogously, the surface of midpiece region can be observed together with the organization of the mitochondria below the plasma membrane, also in comparison with cell presenting anomalies as the spermatozoon with two tails. Indeed in such case SNOM optical images suggest different organization of mitochondria. Finally the organization and arrangement of circumferential ribs and longitudinal column could be followed below the cell surface thanks to SNOM optical transmission images. This capability can be considered very interesting and useful also for the study of morphological and structural characteristics related to other anomalies as for instance: round acrosomeless sperm head that lead to infertility [48,49]; pathologies that involve the mitochondria (number and assembling), which usually lead to reduced sperm motility [50], flagellum abnormalities. [51-54]. Moreover, SNOM may be very helpful also in male contraception investigations, when sperm damages are intentionally provoked [55-57].

In conclusion SNOM can represent a very promising tool to analyze at high magnification (down to nanometer scale) the morphology of cell membrane and underlying structures both in normozoospermia and teratozoospermia without specific staining or harsh sample preparation procedures.

## Methods

### Semen analysis

Ten semen samples from men undergoing routine infertility investigations were obtained. Semen samples were collected by masturbation into sterile containers after 3–6 days of sexual abstinence. After complete liquefaction, routine morphological semen analysis was performed using an optical microscope according to World Health Organization guidelines [4] and the morphology was evaluated by precoloured slides Testsimplets® Waldeck-Muenster/Germany. Five samples showed normozoospermia and five samples displayed teratozoospermia, according to WHO 2010 criteria [4]. In order to test semen quality a leukocyte count was carried out by using standard peroxidase test, as described in the WHO laboratory manual. Leukocytospermia was defined as the presence of >1 × 10^6^ leukocytes per milliliter of semen. For the detection of antisperm antibodies the reagent kit SpermMar® IgG Test from FertiPro N.V. (Sint-Martens-Latem, Belgium) was used.

A very clean sample is fundamental in all kinds of microscopies to obtain high quality images. Particularly in SPM techniques, it is important to avoid that debris attach to the tip during scanning. This could lead to the probe damaging and/or the formation of topographical artifacts. In the case of spermatozoa, the raw semen was processed and purified (washing steps) to get a clean sperm solution free from agglomerated proteins and other cell populations.

### Semen preparation

Semen preparation was performed by Swim-up technique. Such methodology allows obtaining both a spermatozoa selection and a specimen purification. One or more aliquots of 0.5 ml of semen was washed with 1 ml of medium (Quinn’s Advantage Medium w/HEPES, SAGE BioPharma™, Bedminster, NJ, USA, supplemented with 0.5% human serum albumin, SAGE Assisted Reproduction Products™, CooperSurgical, Trumbull, CT, USA) into a 5 ml Falcon conical tube (Becton Dickinson Labware, Meylan, France) and then centrifuged at 300×g for 10 min. The excess of supernatant was discarded and the pellet was resuspended in 0.5 ml of medium. Then, 0.5 ml of medium was gently layered on sperm suspension and the tube was inclinated at an angle of 45° and incubated at 37°C for at least 45 min. The tube was gently set upright and the upper interface was then gently aspirated with a Pasteur pipette. A small aliquot was examined for sperm concentration and sperm motility, the remaining part used for the experiments.

### Sample preparation for SNOM imaging

For further sample cleaning, sperm was washed two times with Dulbecco’s modified phosphate buffered saline (PBS) (Sigma-Aldrich, USA) by centrifugation at 500×g for 5 min and resuspended in PBS (3 × 10^6^ cell/ml). One aliquot of the cell suspension was deposited on poly-L-lysine coated coverslips (18 mm diameter) (Menzler-Gläser) and incubated at 37°C, 5% CO_2_ atmosphere for 1 h to improve the spermatozoa adherence to the substrate. Cells were fixed with 4% paraformaldehyde (PFA) for 15 min at room temperature, washed three times in PBS, two times in water, partially dehydrated (up to 70% ethanol) and allowed to dry before SNOM imaging.

### SNOM set-up

Near-field measurements were performed by using a TriA-SNOM microscope (A.P.E. Research, Trieste, Italy), equipped with a flexure scanning stage with a maximum xyz scan range of 100 μm × 100 μm ×10 μm, with a strain gauge sensors to obtain an absolute positioning of the probe. The TriA-SNOM setup was provided with interchangeable laser sources, coupled with a single mode optical fibre. In this work a laser wavelength of 532 nm was used. This laser source power is 20 mW, but after coupling into the fiber, the radiation power emitted by the aperture of SNOM tip is reduced to 2 μW- 20nW on the sample depending on the aperture diameter and shape of SNOM tip [58]. Beside topography images, SNOM optical reflection and optical transmission signals were simultaneously detected with two photomultipliers (R74000, Hamamatsu Photonics Italia S.r.l., Milano, Italy). For these measurements we used a narrow band-pass interference filter at 532 nm wavelength (full width-half maximum = 10 ± 2 nm). SNOM probe was an aluminum-coated tapered pulled optical fiber with a nominal tip aperture of 50 nm (Lovalite, Troyes, France). Two optical view systems were integrated within the SNOM head to control the probe position and select the scan area. An upper optical vision system was used to monitor probe approach to the sample, and one transmission camera (with interchangeable achromatic objectives) was utilized for a bottom view of the sample. The SNOM images are acquired with: 256×256 pixel and 0.1-0.4 msec acquisition time for each point.

SNOM images were processed by A.P.E. Research SPM control software (A.P.E. Research, Trieste, Italy), and analysed by Gwyddion (free software).

## Additional file

Additional file 1:
**A scan area of head and midpiece region of human sperm cell.** (A) 3D SNOM topography, (B) SNOM reflection and (C) transmission. (D) longitudinal profile along the head. Black arrows in (B) indicate the ends of post-acrosomial part, while red arrows in (C) indicate the ends of the bright layer partially surrounding the head and that includes the acrosome. In (E) cross section profile along the white line in (C) allows evaluating the width of this layer (266 nm).
