# Supplementary material for: The application of scanning near field optical imaging to the study of human sperm morphology
Source: J Nanobiotechnology. 2015 Jan 16;13:2. doi: 10.1186/s12951-014-0061-5 (PMC4302611; doi:10.1186/s12951-014-0061-5)
Supplement: Additional file 1: — A scan area of head and midpiece region of human sperm cell. (A) 3D SNOM topography, (B) SNOM reflection and (C) transmission. (D) longitudinal profile along the head. Black arrows in (B) indicate the ends of post-acrosomial part, while red arrows in (C) indicate the ends of the bright layer partially surrounding the head and that includes the acrosome. In (E) cross section profile along the white line in (C) allows evaluating the width of this layer (266 nm). [file 12951_2014_61_MOESM1_ESM.pdf]

Additional information

The application of Scanning Near Field Optical Imaging to the study of Human Sperm Morphology

L. Andolfi[1], E. Trevisan[2], B. Troian[3], S. Prato[3], R. Boscolo[4], E. Giolo[4], S. Luppi[4], M. Martinelli[4], G. Ricci[4,5] and M. Zweyer[5*]

1. IOM-CNR, Area Science Park, Basovizza, Trieste (Italy)
2. Department of Life Sciences University of Trieste (Italy)
3. A.P.E. Research Srl, AREA Science Park, Basovizza, Trieste (Italy)
4. Institute for Maternal and Child Health, IRCCS Burlo Garofolo Trieste, (Italy)
5. Department of Medicine, Surgery and Health Sciences, University of Trieste, Trieste (Italy)

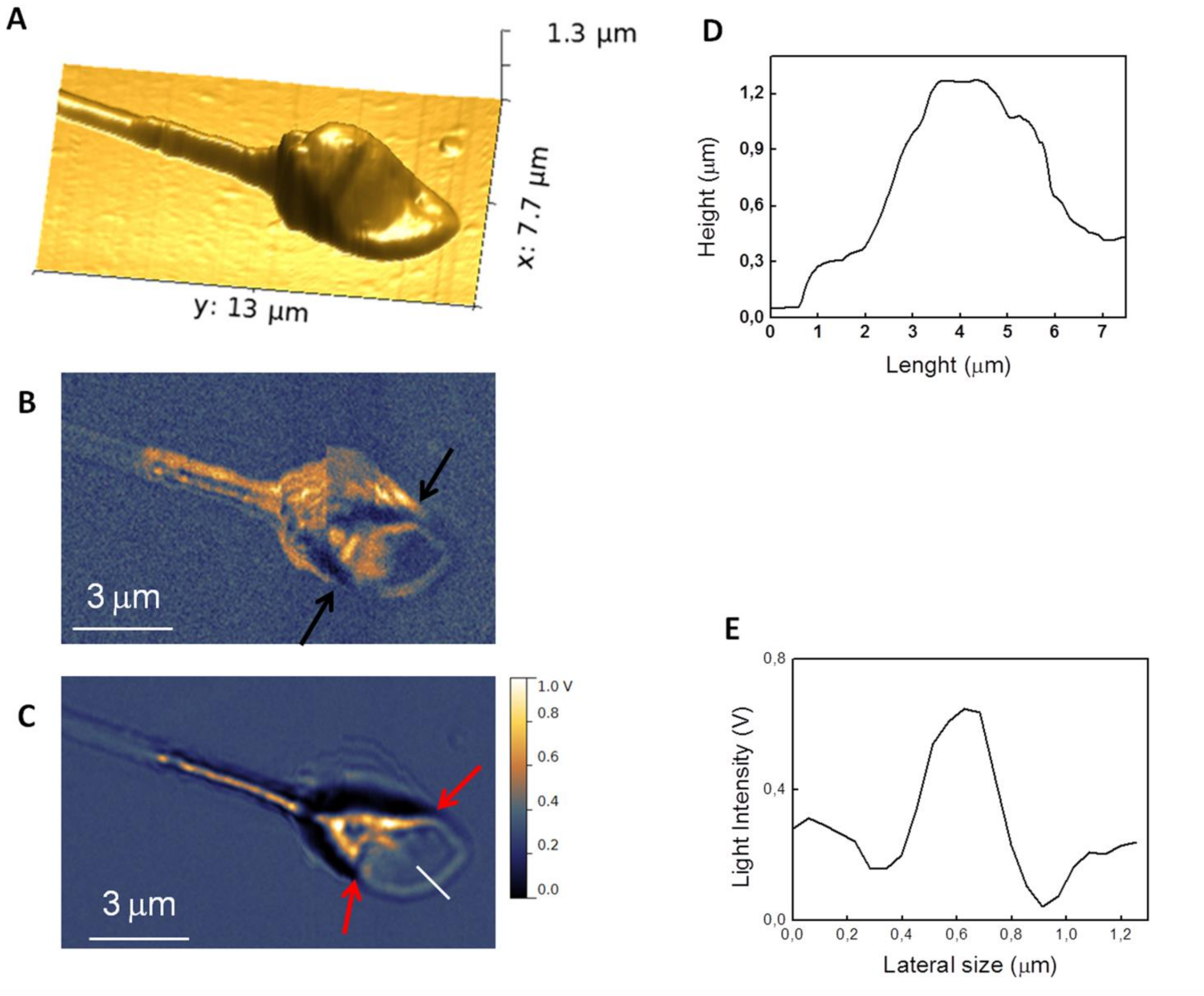

Additional Figure: **A scan area of head and midpiece region of human sperm cell**. (A) 3D SNOM topography, (B) SNOM reflection and (C) transmission. (D) longitudinal profile along the head. Black arrows in (B) indicate the ends of post-acrosomial part, while red arrows in (C) indicate the ends of the bright layer partially surrounding the head and that includes the acrosome. In (E) cross section profile along the white line in (C) allows evaluating the width of this layer (266 nm).
